# KDM2A/B lysine demethylases and their alternative isoforms in development and disease

**DOI:** 10.1080/19491034.2018.1498707

**Published:** 2018-10-08

**Authors:** Tomáš Vacík, Dijana Lađinović, Ivan Raška

**Affiliations:** Institute of Biology and Medical Genetics, First Faculty of Medicine, Charles University and General University Hospital in Prague, Praha 2, Czech Republic

**Keywords:** KDM2A, KDM2B, lysine demethylase, epigenetics, chromatin, alternative isoform, alternative promoter

## Abstract

Aberrant levels of histone modifications lead to chromatin malfunctioning and consequently to various developmental defects and human diseases. Therefore, the proteins bearing the ability to modify histones have been extensively studied and the molecular mechanisms of their action are now fairly well understood. However, little attention has been paid to naturally occurring alternative isoforms of chromatin modifying proteins and to their biological roles. In this review, we focus on mammalian KDM2A and KDM2B, the only two lysine demethylases whose genes have been described to produce also an alternative isoform lacking the N-terminal demethylase domain. These short KDM2A/B-SF isoforms arise through alternative promoter usage and seem to play important roles in development and disease. We hypothesise about the biological significance of these alternative isoforms, which might represent a more common evolutionarily conserved regulatory mechanism.

## Introduction

Epigenetics has become one of the pinnacles of the modern biomedical research and is now known to affect virtually all biological processes ranging from embryogenesis to aging [1–7]. In the nucleosome, the fundamental unit of chromatin, DNA is wrapped around two sets of core histones, H2A, H2B, H3 and H4, whose amino acid residues can be modified by various post-translational modifications such as methylation, acetylation or phosphorylation [8–11]. This creates a large number of potential combinations of various epigenetic marks, each of which has at least one functional consequence frequently distinct from those of other combinations [8,12–14]. This complex network of post-translational modifications of the core histones have been shown to be essential for a large number of cellular processes such as transcription, DNA replication, DNA damage repair, recombination, chromatin structure, cell cycle, and pre-mRNA splicing [1,3,4,8,13,15–17].

Since aberrant levels of histone modifications frequently lead to various developmental defects or diseases including cancer [6,18,19], the proteins bearing the ability to establish (‘writers’), to remove (‘erasers’), or to interpret (“readers) histone modifications have been extensively studied and the molecular mechanisms of their action are now well fairly understood [19–21]. However, little attention has been paid to naturally occurring alternative isoforms of chromatin modifying proteins and to their biological roles. Nevertheless, some interesting alternative isoforms of chromatin modifying enzymes have been already analysed. For example, alternative splicing of the *KDM1A/LSD1* pre-mRNA results in an alternative mRNA isoform that encodes KDM1A/LSD1n, an isoform that demethylates H4K20me1/2 (mono- and di-methylated lysine K20 of histone H4) unlike its canonical KDM1A/LSD1 counterpart that demethylates either H3K4me2 or H3K9me2 in a context dependent manner [22–26]. Interestingly, the same *KDM1A/LSD1* gene can give rise to another alternative KDM1A/LSD1 isoform called LSD1+8a that lacks the H3K4me2 demethylation activity, but retains the H3K9me2 demethylation activity [27]. Methylation of histone H3 represents the most predominant epigenetic modification and is associated various H3 lysines such as K4, K9, K27, K36, K56, K64 or K79 [6,8,9,11,20,28–31]. Methylation or demethylation of a specific histone lysine is mediated by a specific group of lysine methyltransferases or lysine demethylases, respectively, and has specific biological consequences [32–37]. For example, methylation of H3K9, H3K27, or H4K20 is associated with transcriptionally repressed chromatin and its demethylation leads to transcriptional de-repression, whereas methylation of H3K4, H3K36 or H3K79 is associated with transcriptionally active chromatin and its demethylation results in transcriptional repression [20,32,38–43]. Therefore, the three LSD1 isoforms have different impact on transcription of their target genes. By demethylating H3K4me2 or H3K9me2, the canonical LSD1 isoform can function as a transcriptional repressor or activator, respectively, whereas the LSD1+8a and LSD1n isoforms can function only as transcriptional activators by demethylating H3K9me2 and H4K20, respectively [22–27].

In this review we focus on mammalian KDM2A and KDM2B (KDM2A/B), the only two lysine demethylases whose genes have been described to produce both the full length demethylases, KDM2A/B-LF, and also alternative shorter isoforms that lack any demethylation activity, KDM2A/B-SF, but yet seem to also play important roles in various biological processes [44–48]. KDM2A and KDM2B are two closely related lysine demethylases that demethylate histone H3 at lysine K36 [49]. Methylation of H3K36 is indeed an intriguing histone modification since it is involved in a number of various nuclear processes such as transcriptional regulation, gene dosage compensation, pre-mRNA splicing, DNA replication, recombination and DNA damage repair [40,50–53]. As for transcriptional activity, H3K36 methylation is associated with actively transcribed gene bodies to prevent spurious transcription initiations [36,40]. However, H3K36me2 is localised more in the 5´ regions of transcribed genes, whereas H3K36me3 is present predominantly in the 3´ regions [54–56]. Moreover, elevated levels of H3K36me2 have been found to be associated with active gene promoters and removal of this epigenetic mark is associated with transcriptional repression of these promoters [57–64].

## KDM2A and KDM2B: CpG island binding lysine demethylases

**Figure 1.**
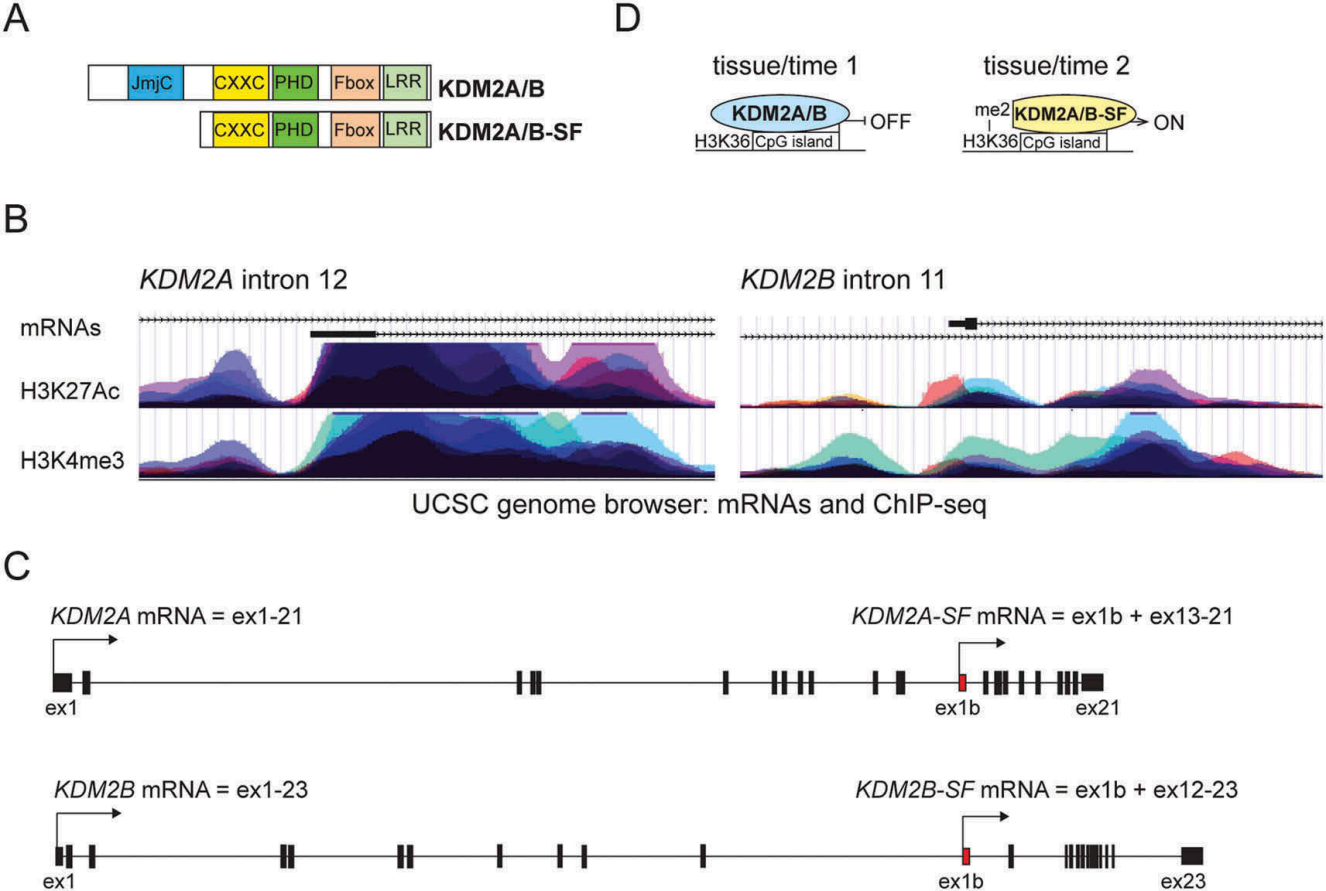
**The lysine demethylases KDM2A and KDM2B and their alternative short isoforms**: (a) The KDM2A and KDM2B lysine demethylases are structurally very similar to each other, bind to unmethylated CpG islands through their CXXC DNA binding domain and demethylate surrounding H3K36me1/2 by their N-terminal Jumonji C demethylase domain (in blue). Their alternative KDM2A/B-SF isoforms lack the N-terminal demethylase domain, but retain all the other functional domains including the DNA binding CXXC domain, through which they can bind to the same DNA regions as KDM2A and KDM2B. (b) Elevated levels of H3K4m3 and H3K27ac at the human short isoform promoter regions. The two UCSC genome browser ENCODE regulation tracks show layered H3K27Ac and H3K4Me3 levels from seven different color coded human cell lines (GM12878, H1-hESC, HSMM, HUVEC, K562, NHEK, NHLF). The higher the peaks, the higher the level of the corresponding histone methylation mark. The UCSC genome browser Genebank mRNA track (mRNAs) shows the first exons of the short *KDM2A/B-SF* mRNA isoforms (black rectangle). (c) The transcription of *KDM2A-SF* and *KDM2B-SF* mRNAs starts with the alternative first exon 1b (in red) in introns 12 and 11, respectively. The exon structure of the *KDM2A-SF* and *KDM2B-SF* mRNAs downstream of exon 1b is identical to that of the full length *KDM2A* and *KDM2B* mRNAs. (d) The putative mechanism of action of the alternative KDM2A/B-SF isoforms. The same promoter is bound and repressed by KDM2A/B in one biological context (tissue/time 1), whereas it is bound and activated by KDM2A/B-SF in another (tissue/time 2), in which the transcriptionally active epigenetic mark H3K36me2 cannot be erased.

The lysine demethylase KDM2A, also known as JHDM1A, FBXL11 or Ndy2, was the first JmjC-domain containing demethylase to be identified [49]. KDM2A and its paralog KDM2B (alias JHDM1B, FBXL10 or Ndy1) contain similar functional domains (Figure1(a)). KDM2A and KDM2B bind to unmethylated CpG islands through their DNA binding CXXC zing finger domain and demethylate mono- and di-methylated lysine K36 of histone H3 (H3K36me1/2) in this area by their N-terminal JmjC demethylase domain [49,62,65–68]. In addition to H3K36me1/2, KDM2B is also able to demethylate two other epigenetic marks of transcriptionally active promoters, H3K4me3 and H3K79me2/3 [31,59,69]. Since demethylation of H3K36me1/2, H3K4me3 and H3K79me2/3 on active gene promoters leads to transcriptional repression, KDM2A and KDM2B function as transcriptional repressors whose depletion leads to de-repression of their target promoters [31,57–64,70–75]. Interestingly, both KDM2A and KDM2B have been shown to demethylate also lysines of non-histone proteins such as the NF-kappaB p65 subunit or beta-catenin [76,77]. Demethylation of the p65 subunit of NF-kappaB at lysines K218 and K221 by KDM2A results in a reduced activity of NF-kappaB and in downregulation of its target genes in mouse fibroblasts [77]. Nuclear beta-catenin, the core component of canonical Wnt signaling [78], is demethylated by both KDM2A and KDM2B, which results in its degradation and consequently in transcriptional repression of canonical Wnt signaling target genes [76]. In human stem cells derived from apical papilla (SCAPs) KDM2A has been shown to affect canonical Wnt signaling in an opposite manner by repressing the promoter of SFRP2, an antagonist of canonical Wnt signaling [61]. In their study, Yu et al. show that knockdown of KDM2A in SCAPs leads to elevated levels of H3K36me2 and H3K4me3 at the *SFRP2* promoter and consequently to upregulation of SFRP2, which results in increased expression of osteo-dentinogenic markers and to enhanced osteo-dentinogenesis [61].

Although plant homeodomains (PHD domains) are generally known to recognise and interact with histone H3 tails [79,80], Zhou et al did not find the KDM2A PHD domain to be able to interact with any of the 600 tested histone variations [81]. However, as opposed to KDM2B, KDM2A is able to interact with heterochromatin proteins HP1 [72], and the KDM2A PHD domain has been shown to be involved in this direct interaction [82]. The HP1 proteins are known to be involved in transcriptional repression by directly interacting with the repressive chromatin mark H3K9me3 [83–88], and the interaction of KDM2A with the HP1 proteins has been suggested to play a role in transcriptional repression of pericentromeric heterochromatin [72]. Furthermore, the ataxia-telangiectasia mutated Ser/Thr kinase has been shown to phosphorylate the KDM2A PHD domain [89], which results in a lower chromatin binding capacity of KDM2A at double strand breaks (DSBs). Consequently, DSBs exhibit higher levels of H3K36me2, which are necessary to attract the DSB repair machinery [89]. KDM2A is further involved in DNA damage repair by interacting with p53-binding protein 1 (53BP1), an essential regulator of DNA DSB repair [90,91]. KDM2A stimulates ubiquitination and stability of 53BP1, promotes recruitment of 53BP1 to DNA breaks, and disruption of the interaction between KDM2A and 53BP1 leads to increased DNA damage-induced genomic instability [91]. As opposed to KDM2A, whose PHD domain does not interact with histones [81], the KDM2B PHD domain has been shown to mediate the interaction with H3K36me2 and H3K4me3 [59]. Interestingly, the KDM2B PHD domain also exhibits an E3 ubiquitin ligase activity [59]. KDM2A and KDM2B are the only members of the JmjC family that contain a C-terminal F-box domain [48,92–94]. As opposed to the KDM2A F-box domain, whose function is still elusive, the KDM2B F-box domain has been shown to mediate protein-protein interactions. For example, using its F-box domain KDM2B interacts with the CUL1-RING ubiquitin ligase complex [95]. The KDM2B F-box and LRR domains are also necessary for interaction with the PcG proteins [96,97].

## KDM2A and KDM2B in development and disease

KDM2A and KDM2B are both highly expressed in mouse embryonic stem cells (ESCs), where they bind to unmethylated CpG island-containing promoters [66,68,98–100]. KDM2B has been shown to form complex with polycomb repressive complex 1 (PRC1) to repress unmethylated CpG island-containing promoters of developmental genes in order to keep mouse ESCs (mESCs) undifferentiated [68,98]. Consistently with these findings, depletion of KDM2B in mESCs induces aberrant differentiation [98,101]. Interestingly, in mESCs the *KDM2B* promoter is directly bound and activated by the pluripotent stem cell factors SOX2 and OCT4 [98]. KDM2B further functions as an anti-adipogenesis factor independently of its N-terminal demethylase domain by dragging the PRC1 repressive complex to the promoters of adipogenesis and cell cycle genes, which results in their repression and in keeping preadipocytes undifferentiated [97]. Similarly, depletion of KDM2A in stem cells from apical papilla (SCAPs) leads to transcriptional de-repression of the stem cell factors NANOG and SOX2, and consequently to differentiation of SCAPs into chondrocytes and adipocytes [102]. KDM2A and KDM2B thus act as stem cell factors that keep various stem cells undifferentiated [60,71,98,101–103], and have been shown to promote generation of induced pluripotent stem cells [100,104,105]. KDM2A is also involved in maintaining cell specific alternative splicing of the *FGFR2* mRNA by forming complex with the PcG protein complex PRC2 and the long noncoding *FGFR2* antisense RNA [106].

Consistent with the high KDM2A expression levels during embryogenesis, the mutant mice lacking the full length KDM2A-LF protein, but retaining the short KDM2A-SF isoform, fail to develop beyond mouse embryonic day 10, show growth retardation and neural tube closure defects [107]. On the other hand, the mice lacking the full length KDM2B-LF protein, but retaining the short KDM2B-SF isoform, exhibit a not fully penetrant phenotype with 40% of the mutants exhibiting retinal coloboma and only 44% of knockouts displaying fatal neural tube closure defects [99]. Although three other studies describe more severe KDM2B knockout phenotypes, these phenotypes are most likely attributable to the loss of both KDM2B-LF and KDM2B-SF as further discussed in the next chapter [96,108,109].

KDM2A exhibits proliferative properties and is upregulated in lung, gastric and breast cancer [47,57,58,74,110]. However, the KDM2A properties are likely to be context dependent since in prostate cancer KDM2A is downregulated [72], and under stress conditions it exhibits anti-proliferative properties by repressing ribosomal RNA (rRNA) genes [70,75]. KDM2A promotes stemness and angiogenesis of breast cancer [64], and promotes silencing of tumor suppressor genes in breast cancer [111]. Overexpression of either KDM2A or KDM2B has been shown to immortalise mouse embryonic fibroblasts in a JmjC domain-dependent process [63,112], and KDM2A and KDM2B have been recently shown to be transcriptionally upregulated in hypoxia [113]. KDM2B is significantly overexpressed in pancreatic ductal adenocarcinoma, ovarian cancer and acute myeloid leukemia [114–116], and drives self-renewal of breast cancer stem cells [117]. Moreover, KDM2B has been recently shown to drive synovial sarcoma [118]. Similarly to KDM2A, the KDM2B properties are also likely to be context dependent since it exhibits anti-proliferative properties by repressing rRNA genes, and its expression is significantly decreased in glioblastoma [69].

Taken together, although KDM2A and KDM2B affect various biological processes through their enzymatic demethylation activities (e.g. direct demethylation of lysines), some processes are affected by these proteins independently of their demethylase domain through their protein partners (e.g. HP1 or PcG proteins) or other catalytic activity (e.g. the ubiquitin ligase activity of the KDM2B PHD domain).

## KDM2A/B-SF: isoforms of lysine demethylases with no demethylation activity

Alternative promoter usage, one of the most widely used mechanisms involved in generating the enormous mammalian proteome complexity, is often responsible for creating protein isoforms lacking their N-terminal domains [119–121]. Since these N-terminal domains frequently have some important function, the canonical isoform and its alternative isoform are then likely to be functionally distinct [46,121–123]. In addition to the still poorly characterised canonical promoters that give rise to the full length *KDM2A/B* mRNAs (KDM2A/B refers to both KDM2A and KDM2B), the *KDM2A* and *KDM2B* loci also contain alternative intronic promoters, through which shorter alternative mRNAs are produced [44,46]. Although the alternative intronic promoters driving the expression of *KDM2A/B-SF* mRNAs (SF stands for short form) have not been characterised yet, they are clearly defined by the presence of the alternative first exons and by the epigenetic profile characteristic for promoters/regulatory elements. For example, the chromatin immunoprecipitation data from various cell types that are publicly available in the UCSC genome browser show that the intronic regions surrounding the alternative first *KDM2A/B-SF* exons exhibit elevated levels of H3K4me3 and H3K27Ac, epigenetic marks associated with active promoter regions [4,124] (Figure1(b)). The alternative *KDM2A-SF* and *KDM2B-SF* mRNAs originate in introns 12 and 11, respectively, and lack the upstream exons that encode the JmjC demethylase domain (Figure 1(c)). The *KDM2A/B-SF* mRNAs thus encode shorter proteins that lack the N-terminal demethylase domain and that are not able to function as demethylases. However, KDM2A/B-SF still retain all the other functional domains including the CXXC, PHD, F-box and LRR domains (Figure 1(a)). As mentioned earlier, many biological processes are mediated by KDM2A and KDM2B independently of their demethylation activity through protein-protein interactions, e.g. interaction with HP1 or PcG proteins [72,82,96,98,101,117,125], or through the ubiquitin ligase activity of the KDM2B PHD domain [59]. KDM2A/B-SF are thus likely to interact with the same proteins as KDM2A/B-LF and to be involved in the same demethylation-independent processes as KDM2A/B-LF. This assumption has already been supported by the following studies. First, both KDM2B isoforms have been indeed shown to be able to interact with PcG proteins and to drag them to CpG islands [96]. Second, KDM2A-SF, which shares the HP1 interaction motif with KDM2A-LF [82], has been indeed shown to complex with the repressive mark H3K9me3 in an HP1a-dependent manner [125]. In our recent study we showed that KDM2A-SF, unlike KDM2A-LF, forms distinct nuclear foci at pericentromeric heterochromatin in an HP1a-dependent way [46].

Since the JmjC demethylase domain of KDM2A/B functions as a 2-oxoglutarate oxygenase [126], its function is dependent on the oxygen levels. Therefore, under low oxygen levels (e.g. hypoxia) the function of the JmjC demethylase domain is compromised and the full length KDM2A/B proteins could then behave as the short KDM2A/B-SF isoforms. In this regard it is also important to mention two more proteins that are related to the KDM2A/B lysine demethylases, but lack any demethylation catalytic activity. Although the JmjC domain of JARID2, a member of the JmjC domain-containing protein family, is not active due to amino acid substitutions in comparison to catalytically active JARID1, its loss-of-function phenotype is embryonically lethal and JARID2 is essential for early development [127]. On the other hand, FBXL19, a member of the F-box family proteins resembles KDM2A/B-SF since it contains the CXXC, PHD, F-box and LRR domains, but completely lacks the JmjC demethylase domain [48,93,94,128]. Despite lacking demethylation activity, FBXL19 plays an important role as a substrate-recognition component of the SCF (SKP1-CUL1-F-box protein)-type E3 ubiquitin ligase complex [93,94], and it has recently been shown to be essential for mouse development [128].

## KDM2A/B-SF in development and disease

Both KDM2B-SF and KDM2A-SF are highly expressed in mESCs and during embryogenesis [46,99]. As opposed to KDM2A-SF, for which knockout mutant mice have not been described yet, the knockout mice that lack KDM2B-SF, but retain KDM2B-LF, have been prepared and analysed [44]. Surprisingly, losing just the short KDM2B-SF isoforms results in severe craniofacial and neural tube defects that are not seen in the KDM2B-LF mutants [44]. The phenotype of losing just KDM2B-LF is milder and not fully penetrant with only 44% of knockouts displaying fatal neural tube closure defects [99]. Although three other studies describe embryonically lethal KDM2B knockout phenotypes, both KDM2B-LF and KDM2B-SF were disrupted in these mutant mice and the described phenotype is thus most likely attributable to the loss of both isoforms [96,108,109]. Based on their different mouse loss-of-function phenotypes, KDM2B-LF and KDM2B-SF seem to be involved in different biological processes. However, similarly to KDM2B-LF, KDM2B-SF is also able to interact with PcG proteins and to attract them to CpG island-containing promoters [96]. Therefore, it is possible that the short and long isoforms also have some redundant roles. In comparison to KDM2A-LF, KDM2A-SF is strongly overexpressed in multiple breast cancer cell lines, exhibits proliferative properties and its knockdown inhibits breast cancer cell growth [47]. Unlike KDM2A-LF that has been shown to repress rRNA genes under stress conditions [70,75], KDM2A-SF has been recently shown promote proliferation by activating rRNA genes in the MCF-7 breast cancer cells [45]. Although the precise molecular mechanism of the KDM2A-SF action is not clear in this case, the authors show that binding of KDM2A-SF to the rDNA promoter results in decreased levels of H4K20me3, a transcriptionally repressive mark, and to activation of the rDNA promoter [45].

## Conclusion

Since the full length KDM2A/B proteins and their shorter alternative KDM2A/B-SF isoforms share the same DNA binding CXXC domain (Figure1(a)), they are likely to bind to the same CpG island-containing DNA regions. However, binding of KDM2A/B-SF, which lack the demethylase domain, cannot lead to demethylation of histone H3 lysines in these regions and to transcriptional repression of associated promoters. Although it needs to be experimentally verified, it is possible that by being unable to demethylate H3K36me2, KDM2A/B-SF would prevent some promoters from being demethylated at H3K36 and from being repressed. Therefore, KDM2A/B-SF would indirectly function as transcriptional activators of these promoters, which is consistent with KDM2A-SF being able to activate the rDNA gene promoter [45]. This assumption is also consistent with the recent study of FBXL19, a member of the KDM2 family that lacks the N-terminal demethylase domain similarly to KDM2A/B-SF. In their study Dimitrova et al. show that FBXL19 is essential for mouse embryogenesis by inducing the expression of developmental genes during ESC differentiation [128]. KDM2A/B-SF might play a role in fine tuning the transcriptional activity of CpG island-containing promoters in various spatio-temporal context. It is possible that certain promoters must be repressed by KDM2A/B-LF in certain cell types and at a certain time-point, one biological context, whereas the same promoters must be activated by KDM2A/B-SF in different cell types and at a different time-point, another biological context (Figure1(d)). Since this context dependent mechanism and the expression of KDM2A/B-SF are likely to be strictly regulated themselves, it will be important to identify the factors regulating both the *KDM2A/B* and *KDM2A/B-SF* promoters. It will be also interesting to analyse whether the *KDM2A/B* and *KDM2A/B-SF* promoters are disrupted by single nucleotide polymorphisms (SNPs) in human diseases or developmental defects. Numerous genome wide association studies have shown that the majority of the SNPs associated with human diseases are located in introns [129,130], and some disease-causing SNPs have even been shown to disrupt transcription factor binding sites in intronic regulatory elements and to affect the expression of the corresponding genes [131,132].

Given some studies on KDM2A and KD2MB have been done without distinguishing the roles of the long isoforms from those of the short isoforms (e.g. the KDM2B-LF and KDM2B-SF double knockout mice [96,108,109], or knockdown of both KDM2A-LF and KDM2A-SF [66,72,91]), it will be necessary to specifically study the roles of just the long or just the short isoforms (e.g. KDM2B-LF specific knockout [99] and KDM2B-SF specific knockout [44]).

As opposed to evolutionarily higher organisms, the fruitfly D. melanogaster contains only one KDM2 lysine demethylase. In Drosophila, the dKdm2 gene encodes a protein that contains the JmjC, CXXC zinc finger, PHD, F-box and LRR domains, and is able to demethylate both H3K36me and H3K4me [133–135]. It will be interesting to determine whether the *KDM2* genes of various lower model organisms (e.g. D. melanogaster, C. elegans) also encode an alternative demethylation inactive isoform. Whether KDM2A/B-SF are the only demethylation inactive alternative isoforms of lysine demethylases or whether this is a more common evolutionarily conserved regulatory mechanism and other alternative isoforms have not been yet identified due to their highly specific spatio-temporal expression pattern, remains to be elucidated in the future.

## References

[1] Sen P, Shah PP, Nativio R, et al Epigenetic Mechanisms of Longevity and Aging. Cell. 2016;166(4):822–839. 27518561 10.1016/j.cell.2016.07.050 PMC5821249

[2] Allshire RC, Madhani HD. Ten principles of heterochromatin formation and function. Nat Rev Mol Cell Biol. 2018;19(4):229-244. 10.1038/nrm.2017.119 PMC6822695 29235574

[3] Xu Q, Xie W Epigenome in Early Mammalian Development: inheritance, Reprogramming and Establishment. Trends Cell Biol. 2018;28(3):237–253. 29217127 10.1016/j.tcb.2017.10.008

[4] Gates LA, Foulds CE, O’Malley BW Histone Marks in the ‘Driver’s Seat’: functional Roles in Steering the Transcription Cycle. Trends Biochem Sci. 2017;42(12):977–989. 29122461 10.1016/j.tibs.2017.10.004 PMC5701853

[5] Atlasi Y, Stunnenberg HG The interplay of epigenetic marks during stem cell differentiation and development. Nat Rev Genet. 2017;18(11):643–658. 28804139 10.1038/nrg.2017.57

[6] Greer EL, Shi Y Histone methylation: a dynamic mark in health, disease and inheritance. Nat Rev Genet. 2012;13(5):343–357. 22473383 10.1038/nrg3173 PMC4073795

[7] Allis CD, Jenuwein T The molecular hallmarks of epigenetic control. Nat Rev Genet. 2016;17(8):487–500. 27346641 10.1038/nrg.2016.59

[8] Kouzarides T Chromatin modifications and their function. Cell. 2007;128(4):693–705. 17320507 10.1016/j.cell.2007.02.005

[9] Henikoff S, Smith MM Histone variants and epigenetics. Cold Spring Harb Perspect Biol. 2015;7(1):a019364. 25561719 10.1101/cshperspect.a019364 PMC4292162

[10] Henikoff S, McKittrick E, Ahmad K Epigenetics, histone H3 variants, and the inheritance of chromatin states. Cold Spring Harb Symp Quant Biol. 2004;69:235–243. 16117654 10.1101/sqb.2004.69.235

[11] Bannister AJ, Kouzarides T Regulation of chromatin by histone modifications. Cell Res. 2011;21(3):381–395. 21321607 10.1038/cr.2011.22 PMC3193420

[12] Berger SL Histone modifications in transcriptional regulation. Curr Opin Genet Dev. 2002;12(2):142–148. 11893486 10.1016/s0959-437x(02)00279-4

[13] Li B, Carey M, Workman JL The role of chromatin during transcription. Cell. 2007;128(4):707–719. 17320508 10.1016/j.cell.2007.01.015

[14] Tessarz P, Kouzarides T Histone core modifications regulating nucleosome structure and dynamics. Nat Rev Mol Cell Biol. 2014;15(11):703–708. 25315270 10.1038/nrm3890

[15] Towbin BD, Gonzalez-Sandoval A, Gasser SM Mechanisms of heterochromatin subnuclear localization. Trends Biochem Sci. 2013;38(7):356–363. 23746617 10.1016/j.tibs.2013.04.004

[16] Cabianca DS, Gasser SM Spatial segregation of heterochromatin: uncovering functionality in a multicellular organism. Nucleus. 2016;7(3):301–307. 27187571 10.1080/19491034.2016.1187354 PMC4991237

[17] Palstra RJ, Grosveld F Transcription factor binding at enhancers: shaping a genomic regulatory landscape in flux. Front Genet. 2012;3:195. 23060900 10.3389/fgene.2012.00195 PMC3460357

[18] Feng L, Zhu J, Chang H, et al The CodY regulator is essential for virulence in Streptococcus suis serotype 2. Sci Rep. 2016;6:26597. 26883762 10.1038/srep21241 PMC4756307

[19] Dawson MA, Kouzarides T Cancer epigenetics: from mechanism to therapy. Cell. 2012;150(1):12–27. 22770212 10.1016/j.cell.2012.06.013

[20] Hyun K, Jeon J, Park K, et al Writing, erasing and reading histone lysine methylations. Exp Mol Med. 2017;49(4):e324. 28450737 10.1038/emm.2017.11 PMC6130214

[21] Helin K, Dhanak D Chromatin proteins and modifications as drug targets. Nature. 2013;502(7472):480–488. 24153301 10.1038/nature12751

[22] Foster CT, Dovey OM, Lezina L, et al Lysine-specific demethylase 1 regulates the embryonic transcriptome and CoREST stability. Mol Cell Biol. 2010;30(20):4851–4863. 20713442 10.1128/MCB.00521-10 PMC2950538

[23] Wang J, Scully K, Zhu X, et al Opposing LSD1 complexes function in developmental gene activation and repression programmes. Nature. 2007;446(7138):882–887. 17392792 10.1038/nature05671

[24] Metzger E, Wissmann M, Yin N, et al LSD1 demethylates repressive histone marks to promote androgen-receptor-dependent transcription. Nature. 2005;437(7057):436–439. 16079795 10.1038/nature04020

[25] Shi Y, Lan F, Matson C, et al Histone demethylation mediated by the nuclear amine oxidase homolog LSD1. Cell. 2004;119(7):941–953. 15620353 10.1016/j.cell.2004.12.012

[26] Wang J, Telese F, Tan Y, et al LSD1n is an H4K20 demethylase regulating memory formation via transcriptional elongation control. Nat Neurosci. 2015;18(9):1256–1264. 26214369 10.1038/nn.4069 PMC4625987

[27] Laurent B, Ruitu L, Murn J, et al A specific LSD1/KDM1A isoform regulates neuronal differentiation through H3K9 demethylation. Mol Cell. 2015;57(6):957–970. 25684206 10.1016/j.molcel.2015.01.010 PMC4369399

[28] Jack APM, Bussemer S, Hahn M, et al H3K56me3 is a novel, conserved heterochromatic mark that largely but not completely overlaps with H3K9me3 in both regulation and localization. PLoS One. 2013;8(2):e51765. 23451023 10.1371/journal.pone.0051765 PMC3579866

[29] Lange UC, Siebert S, Wossidlo M, et al Dissecting the role of H3K64me3 in mouse pericentromeric heterochromatin. Nat Commun. 2013;4:2233. 23903902 10.1038/ncomms3233

[30] Daujat S, Weiss T, Mohn F, et al H3K64 trimethylation marks heterochromatin and is dynamically remodeled during developmental reprogramming. Nat Struct Mol Biol. 2009;16(7):777–781. 19561610 10.1038/nsmb.1629

[31] Kang J-Y, Kim J-Y, Kim K-B, et al KDM2B is a histone H3K79 demethylase and induces transcriptional repression via sirtuin-1-mediated chromatin silencing. The FASEB Journal. 2018. doi:10.1096/fj.201800242R. 10.1096/fj.201800242R 29763382

[32] Shen H, Xu W, Lan F Histone lysine demethylases in mammalian embryonic development. Exp Mol Med. 2017;49(4):e325. 28450736 10.1038/emm.2017.57 PMC6130211

[33] Pedersen MT, Helin K Histone demethylases in development and disease. Trends Cell Biol. 2010;20(11):662–671. 20863703 10.1016/j.tcb.2010.08.011

[34] Shi Y Histone lysine demethylases: emerging roles in development, physiology and disease. Nat Rev Genet. 2007;8(11):829–833. 17909537 10.1038/nrg2218

[35] Klose RJ, Zhang Y Regulation of histone methylation by demethylimination and demethylation. Nat Rev Mol Cell Biol. 2007;8(4):307–318. 17342184 10.1038/nrm2143

[36] Kooistra SM, Helin K Molecular mechanisms and potential functions of histone demethylases. Nat Rev Mol Cell Biol. 2012;13(5):297–311. 22473470 10.1038/nrm3327

[37] Mozzetta C, Boyarchuk E, Pontis J, et al Sound of silence: the properties and functions of repressive Lys methyltransferases. Nat Rev Mol Cell Biol. 2015;16(8):499–513. 26204160 10.1038/nrm4029

[38] Wiles ET, Selker EU H3K27 methylation: a promiscuous repressive chromatin mark. Curr Opin Genet Dev. 2017;43:31–37. 27940208 10.1016/j.gde.2016.11.001 PMC5447479

[39] Eissenberg JC, Shilatifard A Histone H3 lysine 4 (H3K4) methylation in development and differentiation. Dev Biol. 2010;339(2):240–249. 19703438 10.1016/j.ydbio.2009.08.017 PMC3711867

[40] Wagner EJ, Carpenter PB Understanding the language of Lys36 methylation at histone H3. Nat Rev Mol Cell Biol. 2012;13(2):115–126. 22266761 10.1038/nrm3274 PMC3969746

[41] Farooq Z, Banday S, Pandita TK, et al The many faces of histone H3K79 methylation. Mutat Res Rev Mutat Res. 2016;768:46–52. 27234562 10.1016/j.mrrev.2016.03.005 PMC4889126

[42] Nguyen AT, Zhang Y The diverse functions of Dot1 and H3K79 methylation. Genes Dev. 2011;25(13):1345–1358. 21724828 10.1101/gad.2057811 PMC3134078

[43] Nicholson TB, Chen T LSD1 demethylates histone and non-histone proteins. Epigenetics. 2009;4(3):129–132. 19395867 10.4161/epi.4.3.8443

[44] Beh CY, El-Sharnouby S, Chatzipli A, et al. Roles of cofactors and chromatin accessibility in Hox protein target specificity. Epigenetics Chromatin. 2016;9(22):1–9. 26753000 10.1186/s13072-015-0049-x PMC4705621

[45] Okamoto K, Tanaka Y, Tsuneoka M SF-KDM2A binds to ribosomal RNA gene promoter, reduces H4K20me3 level, and elevates ribosomal RNA transcription in breast cancer cells. Int J Oncol. 2017;50(4):1372〓1382. 10.3892/ijo.2017.3908 28350064

[46] Lađinović D, Novotná J, Jakšová S, et al A demethylation deficient isoform of the lysine demethylase KDM2A interacts with pericentromeric heterochromatin in an HP1a-dependent manner. Nucleus. 2017;8(5):563–572. 28816576 10.1080/19491034.2017.1342915 PMC5703260

[47] Liu H, Liu L, Holowatyj A, et al Integrated genomic and functional analyses of histone demethylases identify oncogenic KDM2A isoform in breast cancer. Mol Carcinog. 2016;55(5):977–990. 26207617 10.1002/mc.22341 PMC4724550

[48] Long HK, Blackledge NP, Klose RJ ZF-CxxC domain-containing proteins, CpG islands and the chromatin connection. Biochem Soc Trans. 2013;41(3):727–740. 23697932 10.1042/BST20130028 PMC3685328

[49] Tsukada Y, Fang J, Erdjument-Bromage H, et al Histone demethylation by a family of JmjC domain-containing proteins. Nature. 2006;439(7078):811–816. 16362057 10.1038/nature04433

[50] Kolasinska-Zwierz P, Down T, Latorre I, et al Differential chromatin marking of introns and expressed exons by H3K36me3. Nat Genet. 2009;41(3):376–381. 19182803 10.1038/ng.322 PMC2648722

[51] Schwartz S, Meshorer E, Ast G Chromatin organization marks exon-intron structure. Nat Struct Mol Biol. 2009;16(9):990–995. 19684600 10.1038/nsmb.1659

[52] Pickering T, Hamm JM, Page AF, et al. Cavity-free plasmonic nanolasing enabled by dispersionless stopped light. Nat Commun. 2014;5:4091. 25230337 10.1038/ncomms5972 PMC4199200

[53] Pryde F, Jain D, Kerr A, et al H3 k36 methylation helps determine the timing of cdc45 association with replication origins. PLoS One. 2009;4(6):e5882. 19521516 10.1371/journal.pone.0005882 PMC2690658

[54] Schmähling S, Meiler A, Lee Y, et al. Regulation and function of H3K36 di-methylation by the trithorax-group protein complex AMC. Development. 2018;145(7):1-11. 10.1242/dev.163808 PMC5963871 29540501

[55] Bell O, Wirbelauer C, Hild M, et al Localised H3K36 methylation states define histone H4K16 acetylation during transcriptional elongation in Drosophila. EMBO J. 2007;26(24):4974–4984. 18007591 10.1038/sj.emboj.7601926 PMC2140113

[56] Pokholok DK, Harbison CT, Levine S, et al. Genome-wide map of nucleosome acetylation and methylation in yeast. Cell. 2005;122(4):517–527. 16122420 10.1016/j.cell.2005.06.026

[57] Dhar SS, Alam H, Li N, et al Transcriptional Repression of Histone Deacetylase 3 by the Histone Demethylase KDM2A Is Coupled to Tumorigenicity of Lung Cancer Cells. J Biol Chem. 2014;289(11):7483–7496. 24482232 10.1074/jbc.M113.521625 PMC3953262

[58] Wagner KW, Alam H, Dhar SS, et al KDM2A promotes lung tumorigenesis by epigenetically enhancing ERK1/2 signaling. J Clin Invest. 2013;123(12):5231–5246. 24200691 10.1172/JCI68642 PMC3859406

[59] Janzer A, Stamm K, Becker A, et al The H3K4me3 histone demethylase Fbxl10 is a regulator of chemokine expression, cellular morphology, and the metabolome of fibroblasts. J Biol Chem. 2012;287(37):30984–30992. 22825849 10.1074/jbc.M112.341040 PMC3438931

[60] Du J, Ma Y, Ma P, et al Demethylation of epiregulin gene by histone demethylase FBXL11 and BCL6 corepressor inhibits osteo/dentinogenic differentiation. Stem Cells. 2013;31(1):126–136. 23074094 10.1002/stem.1255

[61] Yu G, Wang J, Lin X, et al Demethylation of SFRP2 by histone demethylase KDM2A regulated osteo-/dentinogenic differentiation of stem cells of the apical papilla. Cell Prolif. 2016;49(3):330–340. 27074224 10.1111/cpr.12256 PMC6496193

[62] He J, Kallin EM, Tsukada Y-I, et al The H3K36 demethylase Jhdm1b/Kdm2b regulates cell proliferation and senescence through p15(Ink4b). Nat Struct Mol Biol. 2008;15(11):1169–1175. 18836456 10.1038/nsmb.1499 PMC2612995

[63] Tzatsos A, Pfau R, Kampranis SC, et al Ndy1/KDM2B immortalises mouse embryonic fibroblasts by repressing the Ink4a/Arf locus. Proc Natl Acad Sci U S A. 2009;106(8):2641–2646. 19202064 10.1073/pnas.0813139106 PMC2650317

[64] Chen J-Y, Li C-F, Chu P-Y, et al Lysine demethylase 2A promotes stemness and angiogenesis of breast cancer by upregulating Jagged1. Oncotarget. 2016;7(19):27689–27710. 27029061 10.18632/oncotarget.8381 PMC5053681

[65] Tsai C-L, Shi Y, Tainer JA How substrate specificity is imposed on a histone demethylase-lessons from KDM2A. Genes Dev. 2014;28(16):1735–1738. 25128493 10.1101/gad.249755.114 PMC4197959

[66] Blackledge NP, Zhou JC, Tolstorukov MY, et al CpG islands recruit a histone H3 lysine 36 demethylase. Mol Cell. 2010;38(2):179–190. 20417597 10.1016/j.molcel.2010.04.009 PMC3098377

[67] Blackledge NP, Klose R CpG island chromatin: a platform for gene regulation. Epigenetics. 2011;6(2):147–152. 20935486 10.4161/epi.6.2.13640 PMC3278783

[68] Farcas AM, Blackledge NP, Sudbery I, et al KDM2B links the Polycomb Repressive Complex 1 (PRC1) to recognition of CpG islands. Elife. 2012;1:e00205. 23256043 10.7554/eLife.00205 PMC3524939

[69] Frescas D, Guardavaccaro D, Bassermann F, et al JHDM1B/FBXL10 is a nucleolar protein that represses transcription of ribosomal RNA genes. Nature. 2007;450(7167):309–313. 17994099 10.1038/nature06255

[70] Tanaka Y, Okamoto K, Teye K, et al JmjC enzyme KDM2A is a regulator of rRNA transcription in response to starvation. EMBO J. 2010;29(9):1510–1522. 20379134 10.1038/emboj.2010.56 PMC2876952

[71] Gao R, Dong R, Du J, et al Depletion of histone demethylase KDM2A inhibited cell proliferation of stem cells from apical papilla by de-repression of p15INK4B and p27Kip1. Mol Cell Biochem. 2013;379(12):115–122. 23559091 10.1007/s11010-013-1633-7

[72] Frescas D, Guardavaccaro D, Kuchay SM, et al KDM2A represses transcription of centromeric satellite repeats and maintains the heterochromatic state. Cell Cycle. 2008;7(22):3539–3547. 19001877 10.4161/cc.7.22.7062 PMC2636745

[73] Yamagishi T, Hirose S, Kondo T Secondary DNA structure formation for Hoxb9 promoter and identification of its specific binding protein. Nucleic Acids Res. 2008;36(6):1965–1975. 18276649 10.1093/nar/gkm1079 PMC2330229

[74] Rizwani W, Schaal C, Kunigal S, et al Mammalian lysine histone demethylase KDM2A regulates E2F1-mediated gene transcription in breast cancer cells. PLoS One. 2014;9(7):e100888. 25029110 10.1371/journal.pone.0100888 PMC4100745

[75] Tanaka Y, Yano H, Ogasawara S, et al Mild Glucose Starvation Induces KDM2A-Mediated H3K36me2 Demethylation through AMPK To Reduce rRNA Transcription and Cell Proliferation. Mol Cell Biol. 2015;35(24):4170–4184. 26416883 10.1128/MCB.00579-15 PMC4648824

[76] Lu L, Gao Y, Zhang Z, et al Kdm2a/b Lysine Demethylases Regulate Canonical Wnt Signaling by Modulating the Stability of Nuclear β-Catenin. Dev Cell. 2015;33(6):660–674. 26004508 10.1016/j.devcel.2015.04.006

[77] Lu T, Jackson MW, Wang B, et al Regulation of NF-kappaB by NSD1/FBXL11-dependent reversible lysine methylation of p65. Proc Natl Acad Sci U S A. 2010;107(1):46–51. 20080798 10.1073/pnas.0912493107 PMC2806709

[78] Nusse R, Clevers H Wnt/beta-Catenin Signaling, Disease, and Emerging Therapeutic Modalities. Cell. 2017;169(6):985–999. 28575679 10.1016/j.cell.2017.05.016

[79] Musselman CA, Kutateladze TG Handpicking epigenetic marks with PHD fingers. Nucleic Acids Res. 2011;39(21):9061–9071. 21813457 10.1093/nar/gkr613 PMC3241642

[80] Taverna SD, Li H, Ruthenburg AJ, et al How chromatin-binding modules interpret histone modifications: lessons from professional pocket pickers. Nat Struct Mol Biol. 2007;14(11):1025–1040. 17984965 10.1038/nsmb1338 PMC4691843

[81] Zhou JC, Blackledge NP, Farcas AM, et al Recognition of CpG island chromatin by KDM2A requires direct and specific interaction with linker DNA. Mol Cell Biol. 2012;32(2):479–489. 22083960 10.1128/MCB.06332-11 PMC3255781

[82] Borgel J, Tyl M, Schiller K, et al KDM2A integrates DNA and histone modification signals through a CXXC/PHD module and direct interaction with HP1. Nucleic Acids Res. 2017;45(3):1114–1129. 28180290 10.1093/nar/gkw979 PMC5388433

[83] Eissenberg JC, Elgin SCR HP1a: a structural chromosomal protein regulating transcription. Trends Genet. 2014;30(3):103–110. 24555990 10.1016/j.tig.2014.01.002 PMC3991861

[84] Canzio D, Larson A, Narlikar GJ Mechanisms of functional promiscuity by HP1 proteins. Trends Cell Biol. 2014;24(6):377–386. 24618358 10.1016/j.tcb.2014.01.002 PMC4077871

[85] Kwon SH, Workman JL The changing faces of HP1: from heterochromatin formation and gene silencing to euchromatic gene expression: HP1 acts as a positive regulator of transcription. Bioessays. 2011;33(4):280–289. 21271610 10.1002/bies.201000138

[86] Maison C, Almouzni G HP1 and the dynamics of heterochromatin maintenance. Nat Rev Mol Cell Biol. 2004;5(4):296–304. 15071554 10.1038/nrm1355

[87] Bannister AJ, Zegerman P, Partridge JF, et al Selective recognition of methylated lysine 9 on histone H3 by the HP1 chromo domain. Nature. 2001;410(6824):120–124. 11242054 10.1038/35065138

[88] Lachner M, O’Carroll D, Rea S, et al Methylation of histone H3 lysine 9 creates a binding site for HP1 proteins. Nature. 2001;410(6824):116–120. 11242053 10.1038/35065132

[89] Cao LL, Wei F, Du Y, et al. ATM-mediated KDM2A phosphorylation is required for the DNA damage repair. Oncogene. 2016;35(3):301-313. 10.1038/onc.2015.81 25823024

[90] Panier S, Boulton SJ Double-strand break repair: 53BP1 comes into focus. Nat Rev Mol Cell Biol. 2014;15(1):7–18. 24326623 10.1038/nrm3719

[91] Bueno MTD, Baldascini M, Richard S, et al Recruitment of lysine demethylase 2A to DNA double strand breaks and its interaction with 53BP1 ensures genome stability. Oncotarget. 2018;9(22):15915–15930. 29662616 10.18632/oncotarget.24636 PMC5882307

[92] Kipreos ET, Pagano M The F-box protein family. Genome Biol. 2000;1(5):REVIEWS3002. 11178263 10.1186/gb-2000-1-5-reviews3002 PMC138887

[93] Shen J, Spruck C F-box proteins in epigenetic regulation of cancer. Oncotarget. 2017;8(66):110650–110655. 29299176 10.18632/oncotarget.22469 PMC5746411

[94] Randle SJ, Laman H F-box protein interactions with the hallmark pathways in cancer. Semin Cancer Biol. 2016;36:3–17. 26416465 10.1016/j.semcancer.2015.09.013

[95] Han X-R, Zha Z, Yuan H-X, et al KDM2B/FBXL10 targets c-Fos for ubiquitylation and degradation in response to mitogenic stimulation. Oncogene. 2016;35(32):4179–4190. 10.1038/onc.2015.482 PMC4931990 26725323

[96] Blackledge NP, Farcas AM, Kondo T, et al Variant PRC1 Complex-Dependent H2A Ubiquitylation Drives PRC2 Recruitment and Polycomb Domain Formation. Cell. 2014;157(6):1445–1459. 24856970 10.1016/j.cell.2014.05.004 PMC4048464

[97] Inagaki T, Iwasaki S, Matsumura Y, et al The FBXL10/KDM2B scaffolding protein associates with novel polycomb repressive complex-1 to regulate adipogenesis. J Biol Chem. 2015;290(7):4163–4177. 25533466 10.1074/jbc.M114.626929 PMC4326826

[98] He J, Shen L, Wan M, et al Kdm2b maintains murine embryonic stem cell status by recruiting PRC1 complex to CpG islands of developmental genes. Nat Cell Biol. 2013;15(4):373–384. 23502314 10.1038/ncb2702 PMC4078788

[99] Fukuda T, Tokunaga A, Sakamoto R, et al Fbxl10/Kdm2b deficiency accelerates neural progenitor cell death and leads to exencephaly. Mol Cell Neurosci. 2011;46(3):614–624. 21220025 10.1016/j.mcn.2011.01.001

[100] Liang G, He J, Zhang Y Kdm2b promotes induced pluripotent stem cell generation by facilitating gene activation early in reprogramming. Nat Cell Biol. 2012;14(5):457–466. 22522173 10.1038/ncb2483 PMC3544197

[101] Wu X, Johansen JV, Helin K Fbxl10/Kdm2b recruits polycomb repressive complex 1 to CpG islands and regulates H2A ubiquitylation. Mol Cell. 2013;49(6):1134–1146. 23395003 10.1016/j.molcel.2013.01.016

[102] Dong R, Yao R, Du J, et al Depletion of histone demethylase KDM2A enhanced the adipogenic and chondrogenic differentiation potentials of stem cells from apical papilla. Exp Cell Res. 2013;319(18):2874–2882. 23872478 10.1016/j.yexcr.2013.07.008

[103] Wang Z, Gearhart MD, Lee Y-W, et al A Non-canonical BCOR-PRC1.1 Complex Represses Differentiation Programs in Human ESCs. Cell Stem Cell. 2018;22(2): 235–251. e9. 29337181 10.1016/j.stem.2017.12.002 PMC5797497

[104] Zhou Z, Yang X, He J, et al Kdm2b Regulates Somatic Reprogramming through Variant PRC1 Complex-Dependent Function. Cell Rep. 2017;21(8):2160–2170. 29166607 10.1016/j.celrep.2017.10.091

[105] Wang T, Chen K, Zeng X, et al The histone demethylases Jhdm1a/1b enhance somatic cell reprogramming in a vitamin-C-dependent manner. Cell Stem Cell. 2011;9(6):575–587. 22100412 10.1016/j.stem.2011.10.005

[106] Gonzalez I, Munita R, Agirre E, et al A lncRNA regulates alternative splicing via establishment of a splicing-specific chromatin signature. Nat Struct Mol Biol. 2015;22(5):370–376. 25849144 10.1038/nsmb.3005 PMC6322542

[107] Kawakami E, Tokunaga A, Ozawa M, et al The histone demethylase fbxl11/kdm2a plays an essential role in embryonic development by repressing cell-cycle regulators. Mech Dev. 2015;135:31–42. 25463925 10.1016/j.mod.2014.10.001

[108] Andricovich J, Kai Y, Peng W, et al Histone demethylase KDM2B regulates lineage commitment in normal and malignant hematopoiesis. J Clin Invest. 2016;126(3):905–920. 26808549 10.1172/JCI84014 PMC4767361

[109] Boulard M, Edwards JR, Bestor TH FBXL10 protects Polycomb-bound genes from hypermethylation. Nat Genet. 2015;47(5):479–485. 25848754 10.1038/ng.3272

[110] Huang Y, Liu Y, Yu L, et al Histone demethylase KDM2A promotes tumor cell growth and migration in gastric cancer. Tumour Biol. 2015;36(1):271–278. 25245333 10.1007/s13277-014-2630-5

[111] Chen J-Y, Luo C-W, Lai Y-S, et al Lysine demethylase KDM2A inhibits TET2 to promote DNA methylation and silencing of tumor suppressor genes in breast cancer. Oncogenesis. 2017;6(8):e369. 28785073 10.1038/oncsis.2017.71 PMC5608919

[112] Pfau R, Tzatsos A, Kampranis SC, et al Members of a family of JmjC domain-containing oncoproteins immortalise embryonic fibroblasts via a JmjC domain-dependent process. Proc Natl Acad Sci U S A. 2008;105(6):1907–1912. 18250326 10.1073/pnas.0711865105 PMC2538857

[113] Batie M, Druker J, D’Ignazio L, et al KDM2 Family Members are Regulated by HIF-1 in Hypoxia. Cells. 2017;6(1):8–16. 10.3390/cells6010008 PMC5371873 28304334

[114] Tzatsos A, Paskaleva P, Ferrari F, et al KDM2B promotes pancreatic cancer via Polycomb-dependent and -independent transcriptional programs. J Clin Invest. 2013;123(2):727–739. 23321669 10.1172/JCI64535 PMC3561797

[115] Kuang Y, Lu F, Guo J, et al Histone demethylase KDM2B upregulates histone methyltransferase EZH2 expression and contributes to the progression of ovarian cancer in vitro and in vivo. Onco Targets Ther. 2017;10:3131–3144. 28706445 10.2147/OTT.S134784 PMC5495092

[116] He J, Nguyen AT, Zhang Y KDM2b/JHDM1b, an H3K36me2-specific demethylase, is required for initiation and maintenance of acute myeloid leukemia. Blood. 2011;117(14):3869–3880. 21310926 10.1182/blood-2010-10-312736 PMC3083299

[117] Kottakis F, Foltopoulou P, Sanidas I, et al NDY1/KDM2B functions as a master regulator of Polycomb complexes and controls self-renewal of breast cancer stem cells. Cancer Res. 2014;74(14):3935–3946. 24853546 10.1158/0008-5472.CAN-13-2733 PMC4454481

[118] Banito A, Li X, Laporte AN, et al The SS18-SSX Oncoprotein Hijacks KDM2B-PRC1.1 to Drive Synovial Sarcoma. Cancer Cell. 2018;33(3): 527–541. e8. 29502955 10.1016/j.ccell.2018.01.018 PMC5881394

[119] De Klerk E, T Hoen PA Alternative mRNA transcription, processing, and translation: insights from RNA sequencing. Trends Genet. 2015;31(3):128–139. 25648499 10.1016/j.tig.2015.01.001

[120] Davuluri RV, Suzuki Y, Sugano S, et al The functional consequences of alternative promoter use in mammalian genomes. Trends Genet. 2008;24(4):167–177. 18329129 10.1016/j.tig.2008.01.008

[121] Vacik T, Raska I Alternative intronic promoters in development and disease. Protoplasma. 2017;254(3):1201–1206. 28078440 10.1007/s00709-016-1071-y

[122] Vacik T, Stubbs JL, Lemke G A novel mechanism for the transcriptional regulation of Wnt signaling in development. Genes Dev. 2011;25(17):1783–1795. 21856776 10.1101/gad.17227011 PMC3175715

[123] Vacik T, Lemke G Dominant-negative isoforms of Tcf/Lef proteins in development and disease. Cell Cycle. 2011;10(24):4199–4200. 22157225 10.4161/cc.10.24.18465

[124] Kent WJ, Sugnet CW, Furey TS, et al The human genome browser at UCSC. Genome Res. 2002;12(6):996–1006. 12045153 10.1101/gr.229102 PMC186604

[125] Bartke T, Vermeulen M, Xhemalce B, et al Nucleosome-interacting proteins regulated by DNA and histone methylation. Cell. 2010;143(3):470–484. 21029866 10.1016/j.cell.2010.10.012 PMC3640253

[126] Accari SL, Fisher PR Emerging Roles of JmjC Domain-Containing Proteins. Int Rev Cell Mol Biol. 2015;319:165–220. 26404469 10.1016/bs.ircmb.2015.07.003

[127] Landeira D, Fisher AG Inactive yet indispensable: the tale of Jarid2. Trends Cell Biol. 2011;21(2):74–80. 21074441 10.1016/j.tcb.2010.10.004 PMC3034028

[128] Dimitrova E, Kondo T, Feldmann A, et al FBXL19 recruits CDK-Mediator to CpG islands of developmental genes priming them for activation during lineage commitment. Elife. 2018;7:1-27. 10.7554/eLife.37084 PMC5997449 29809150

[129] Li MJ, Liu Z, Wang P, et al GWASdb v2: an update database for human genetic variants identified by genome-wide association studies. Nucleic Acids Res. 2016;44:D869D876. 26615194 10.1093/nar/gkv1317 PMC4702921

[130] Tak YG, Farnham PJ Making sense of GWAS: using epigenomics and genome engineering to understand the functional relevance of SNPs in non-coding regions of the hum,an genome. Epigenetics Chromatin. 2015;8:57. 26719772 10.1186/s13072-015-0050-4 PMC4696349

[131] Farh KK-H, Marson A, Zhu J, et al Genetic and epigenetic fine mapping of causal autoimmune disease variants. Nature. 2015;518(7539):337–343. 25363779 10.1038/nature13835 PMC4336207

[132] Oldridge DA, Wood AC, Weichert-Leahey N, et al Genetic predisposition to neuroblastoma mediated by a LMO1 super-enhancer polymorphism. Nature. 2015;528(7582):418–421. 26560027 10.1038/nature15540 PMC4775078

[133] Zheng Y, Hsu F-N, Xu W, et al A developmental genetic analysis of the lysine demethylase KDM2 mutations in Drosophila melanogaster. Mech Dev. 2014;133:36–53. 25016215 10.1016/j.mod.2014.06.003 PMC4177295

[134] Kavi HH, Birchler JA Drosophila KDM2 is a H3K4me3 demethylase regulating nucleolar organization. BMC Res Notes. 2009;2:217. 19852816 10.1186/1756-0500-2-217 PMC2771041

[135] Lagarou A, Mohd-Sarip A, Moshkin YM, et al dKDM2 couples histone H2A ubiquitylation to histone H3 demethylation during Polycomb group silencing. Genes Dev. 2008;22(20):2799–2810. 18923078 10.1101/gad.484208 PMC2569881

